# *RNF213*-Associated Vascular Disease: A Concept Unifying Various Vasculopathies

**DOI:** 10.3390/life12040555

**Published:** 2022-04-08

**Authors:** Takahiro Hiraide, Hisato Suzuki, Mizuki Momoi, Yoshiki Shinya, Keiichi Fukuda, Kenjiro Kosaki, Masaharu Kataoka

**Affiliations:** 1 Department of Cardiology, Keio University School of Medicine, Shinjuku-ku, Tokyo 160-8582, Japan; t.hiraide.a6@keio.jp (T.H.); m.momoi@keio.jp (M.M.); y.shinya@keio.jp (Y.S.); kfukuda@a2.keio.jp (K.F.); 2 Center for Medical Genetics, Keio University School of Medicine, Tokyo 160-8582, Japan; hsuzuki@keio.jp (H.S.); kkosaki@keio.jp (K.K.); 3 The Second Department of Internal Medicine, University of Occupational and Environmental Health, Kitakyushu 807-8555, Japan

**Keywords:** *RNF213*, pulmonary arterial hypertension, moyamoya disease, gene-associated vasculopathy

## Abstract

The ring finger protein 213 gene (*RNF213*) encodes a 590 kDa protein that is thought to be involved in angiogenesis. This gene was first recognized as a vasculopathy-susceptibility locus through genome-wide association studies undertaken in a Japanese population, demonstrating that heterozygotes for *RNF213* p.Arg4810Lys (c.14429G>A, rs112735431) had a greatly increased risk of moyamoya disease. The association of *RNF213* p.Arg4810Lys as a susceptibility variant of moyamoya disease was reproduced in Korean and Chinese individuals and, later, in Caucasians. Variants of the *RNF213* gene have been linked to a number of vascular diseases such as moyamoya disease, intracranial major artery stenosis, pulmonary arterial hypertension, and peripheral pulmonary artery stenosis, and have also been associated with co-occurrent diseases and vascular disease in different organs. Based on the findings that we have reported to date, our paper proposes a new concept of “*RNF213*-associated vascular disease” to unify these conditions with the aim of capturing patients with multiple diseases but with a common genetic background. This concept will be highly desirable for clarifying all of the diseases in the *RNF213*-associated vascular disease category by means of global epidemiological investigations because of the possibility of such diseases appearing asymptomatically in some patients.

The ring finger protein 213 gene (*RNF213*; NM_001256071.2) encodes a 590 kDa protein that contains a really interesting new gene (RING) finger domain with E3 ubiquitin-protein ligase activity and 2 regions of ATPase-associated domains. This gene was first recognized as a vasculopathy-susceptibility locus in 2011 through the genome-wide linkage analysis of Japanese families with moyamoya disease, which demonstrated that heterozygotes for *RNF213* p.Arg4810Lys (c.14429G>A, rs112735431) might be susceptible to moyamoya disease [1]. Moyamoya disease is characterized by progressive stenosis and the subsequent occlusion of the intracranial arteries. Cerebral angiography shows compensatory fragile collateral networks, giving a smoky impression described as “moyamoya” in Japanese. A genome-wide association study (GWAS) analysis in Japanese patients with moyamoya disease demonstrated that 95% of familial patients and 46 out of 63 non-familial patients (73%) had the *RNF213* p.Arg4810Lys variant, and this variant conferred a greatly increased risk of moyamoya disease (odds ratio, 190.8) [2]. The association of *RNF213* p.Arg4810Lys as a susceptibility variant of moyamoya disease was reproduced in Korean and Chinese individuals [3,4]. A previous meta-analysis of Asian patients with moyamoya disease demonstrated that Chinese patients showed a lower allele frequency for the *RNF213* p.Arg4810Lys variant compared to Japanese and Korean patients, and the *RNF213* p.Ala5021Val variant was also associated with the development of moyamoya disease in the Chinese population [4]. Although the *RNF213* p.Arg4810Lys variant was hardly detected, several pathogenetic variants of *RNF213* have been identified in Caucasian patients with moyamoya disease [5]. The whole-exome sequencing of 68 Caucasian patients with moyamoya disease or moyamoya syndrome demonstrated that rare missense variants of *RNF213* were associated with the development of moyamoya disease (odds ratio, 2.24); moreover, a hotspot of rare variants was identified for the 6.2kb C-terminal region near the RING-finger domain. Familial patients with moyamoya disease showed a strong association with the *RNF213* pathogenetic variants (odds ratio, 4.54), consistent with the case of the Asian population [5]. The variants of *RNF213*, especially the p.Arg4810Lys variant, have been identified as a key moderator of development in moyamoya disease.

In 2016, our group identified 2 patients with a homozygous mutation of *RNF213*, p.Arg4810Lys, who developed moyamoya disease and severe pulmonary hypertension caused by peripheral pulmonary artery stenosis. This is characterized by progressive stenosis and the subsequent occlusion of the pulmonary arteries, so the pathological condition macroscopically resembles moyamoya disease in pulmonary vessels [6]. Therefore, we first conceptualized that a genetic abnormality in *RNF213* causes systemic vasculopathy, which we named “*RNF213*-associated vascular disease” (Figure 1). This concept was consistent with a report that the homozygous *RNF213* p.Arg4810Lys variant was associated with intracranial and extracranial vasculopathy, including adulthood-onset peripheral pulmonary artery stenosis in segmental or subsegmental arteries [7]. We also reported one family with the *RNF213* p.Arg4810Lys variant in which the mother developed pulmonary arterial hypertension (PAH), and her daughter had moyamoya disease. Notably, the magnetic resonance angiography of the mother’s brain did not exhibit moyamoya disease, and her daughter had no evidence of PAH [6]. Our notion was further reinforced by our own discovery in 2018 that the allele frequency of the heterozygous *RNF213* p.Arg4810Lys variant was significantly higher in 76 Japanese patients with PAH, compared to that in 79 Japanese supercentenarians who had lived to over 110 years old and never experienced significant health problems [8]. In 2020, we also clarified that the *RNF213* p.Arg4810Lys variant was present in approximately 8% of patients with idiopathic PAH, and these idiopathic PAH patients with the *RNF213* p.Arg4810Lys variant responded poorly to current combination therapy using PAH-specific vasodilators compared to those who carried mutations in the bone morphogenic protein receptor type 2 (*BMPR2*) gene, which is the most common pathogenic gene in patients with PAH [9]. A Kaplan–Meier analysis regarding death or lung transplantation demonstrated that the event-free rate was significantly lower in PAH patients with the *RNF213* p.Arg4810Lys variant than in those with *BMPR2* mutations [9]. Multivariate analysis identified that the *RNF213* p.Arg4810Lys variant was an independent predictor of worse clinical outcomes in patients with PAH. Several other variants of *RNF213* have also been identified in patients with idiopathic PAH, but these variants were not independently associated with the poor clinical outcomes. Representative images of the lung specimens from patients with the *RNF213* p.Arg4810Lys variant demonstrated microscopically progressive stenosis, concentric remodeling, and subsequent occlusion of the pulmonary capillaries and veins as well as pulmonary arteries, which is not typical for patients with severe idiopathic PAH with *BMPR2* mutations. Since the current therapy for PAH targets vascular smooth muscle contraction in pulmonary arteries, venous lesions may be associated with a poor reactivity for vasodilators and worse clinical outcomes [9]. Early consideration of lung transplantation may be a therapeutic option for patients with PAH who have the heterozygous *RNF213* p.Arg4810Lys variant.

The allele frequency of this “hotspot” variant, p.Arg4810Lys, is 0.7681% in the general Japanese population, according to the Integrative Japanese Genome Variation Database (the genome cohort study of the Tohoku Medical Megabank Organization); this demonstrates a higher frequency in East Asian populations compared with non-Asian populations. However, importantly, several other *RNF213* variants were found in non-Asian populations with moyamoya disease [10,11]. Moreover, some heterozygous *RNF213* mutations are frequently observed in several vascular diseases including intracranial major artery stenosis/occlusion (ICASO) [12,13], intracranial artery aneurysm/dissection [14,15], coronary artery disease [16], and thoracic aortic aneurysm/dissection [17] (Figure 1, highlighted in pink) in both Asian and non-Asian populations. A meta-analysis study with whole-exome sequencing of 1778 Asian patients with ICASO demonstrated that several *RNF213* pathogenetic variants, including the p.Arg4810Lys variant, significantly increased the risk of ICASO [12]. Whole-exome sequencing of 233 French–Canadian patients with intracranial aneurysms identified 17 deleterious rare *RNF213* variants, and the *RNF213* single-nucleotide polymorphism rs6565666 was significantly associated with the occurrence of intracranial aneurysms in the French–Canadian population [15]. A recent study of 702 Han Chinese patients with sporadic aortic dissection revealed that pathogenetic or likely pathogenetic *RNF213* variants were identified in 26 patients (3.7%), and 7 of them did not have any pathogenetic variations in genes known to be associated with aortic dissection, such as *FBN1*, *ACTA2*, and *MYH11*. Transcriptome analysis revealed that the messenger RNA expression of *RNF213* was linked with that of *FBN1*, the most frequent pathogenetic gene in aortic dissection, suggesting that reduced *RNF213* expression might be associated with the interruption of aortic development [17]. Therefore, *RNF213*-associated vascular disease is not only an endemic concept for Asian populations but is also important in non-Asian populations such as Caucasians [4].

Furthermore, co-occurrent cases of moyamoya disease with retinal vessel occlusion [18,19], carotid artery stenosis [20], and renal artery stenosis [21,22,23] have been identified, suggesting that these vascular diseases may be associated with *RNF213* variants (Figure 1, highlighted in black). Out of 86 consecutive patients with Japanese moyamoya disease, abdominal aortographies were identified 6 patients (7%) with renal artery stenosis and 1 patient (1%) with a renal artery aneurysm. Notably, there was no significant association between the presence of renal artery lesions and cerebral artery phenotypes [22]. Another case report describes a patient with moyamoya disease who developed renal artery stenosis during long-term follow-up [24]. These findings imply the possibility that these vascular diseases are disseminated in time and space. Furthermore, peripheral arteriopathy, which resembles the phenotype of Takayasu’s arteritis, has been identified in some patients with moyamoya disease [25,26]. Takayasu’s arteritis causes systemic granulomatous vascular inflammation, massive intimal fibrosis, and vascular stenosis/occlusion in large vessels, predominantly the aorta and main branches. Furthermore, several studies have shown that patients with Takayasu’s arteritis are at an increased risk of pulmonary arterial vasculopathy [27,28]. Although the exact etiology of Takayasu’s arteritis is unknown, both genetic and environmental factors are thought to be involved. These findings suggest that some cases of peripheral arteriopathy or Takayasu’s arteritis might be associated with *RNF213* variants (Figure 1, highlighted in gray).

Previous studies have demonstrated that *RNF213*-knockout zebrafish have abnormal vessel sprouting [1] and that the overexpression of the *RNF213* p.Arg4810Lys variant in endothelial cells causes abnormal tubular formation, whereas the silencing of *RNF213* does not [29]. These studies raise the possibility that *RNF213* is associated with the regulation of angiogenesis and the *RNF213* p.Arg4810Lys variant has gain-of-function activity. Hence, the *RNF213* p.Arg4810Lys variant does not appear to be a mere innocent bystander identified by GWAS, but rather a pathogenic variant responsible for vascular phenotypes; thus, *RNF213* suppression, if permissible, may represent a novel therapeutic approach to treating systemic vasculopathy. In a murine model, however, transgenic mice with *RNF213* knockout [30] or the *RNF213* point missense mutation p.Arg4828Lys, which corresponds to the human p.Arg4810Lys variant [31], did not demonstrate the phenotypes mimicking moyamoya disease and other vasculopathies in a normoxic environment. Since the penetrance of the *RNF213* p.Arg4810Lys variant for moyamoya disease and PAH is quite low in human studies, the *RNF213* p.Arg4810Lys variant is considered a susceptibility gene variant, and carriers may need a second-hit factor to develop vasculopathy. Further study is warranted to elucidate the additional factors accelerating development in *RNF213*-associated vascular disease.

The molecular mechanism of *RNF213* in the development of vessel remodeling has previously been investigated. A study demonstrated that *Rnf213* attenuated non-canonical WNT/calcium signaling via the degradation of filamin A and nuclear factor of activated T cells, resulting in impaired vessel remodeling in R-spondin 3-deficient mice [32]. Sarkar P et al. identified that *RNF213* was associated with tumor necrosis factor alpha-mediated inflammation in both macrophages and adipocytes, resulting in obesity, increased insulin resistance, and vascular remodeling [33]. These studies highlighted the close association between *RNF213* and systemic vascular inflammation, which is consistent with a clinical report that plasma samples from patients with moyamoya disease showed elevated serum levels of matrix metalloproteinases 9, monocyte chemoattractant protein-1, proinflammatory interleukins, and vascular endothelial growth factor [34]. Moreover, another study demonstrated that the *RNF213*-encoded protein regulated the maintenance of cellular fat storage via increasing the number of lipid droplets by interfering with lipolysis. Rare variants identified in Caucasian patients with moyamoya disease, such as p.Cys3997Tyr and p.His4014Asn, were associated with an abnormal lipid metabolism, whereas variants in Asian patients, such as p.Arg4810Lys and p.Asp4013Asn, were not [35]. Furthermore, *RNF213* was associated with an excessive level of saturated fatty acids, and the depletion of *RNF213* protected against palmitate-mediated lipotoxicity [36]. A recent study in Salmonella demonstrated that the *RNF213*-encoded protein was necessary for the ubiquitylation of lipopolysaccharide to maintain the bacterial ubiquitin coat, and a defect in *RNF213* caused impaired autonomous immunity in cells infected by *Salmonella* [37]. These results highlight the importance of *RNF213* E3 ubiquitin ligase activity in lipid metabolism and immune-system regulation. Further studies are warranted to elucidate the precise molecular mechanism of the development of *RNF213*-associated vascular disease.

Several questions need to be answered with regard to “*RNF213*-associated vascular disease”. First, how relevant are *RNF213* variants in non-Asian populations? Some studies have shown relevance, but these have been limited in population size and diversity. Second, why do some *RNF213* heterozygotic variants never lead to the development of any vasculopathy? A mouse model provided a clue to the answer to this question. Transgenic mice with a vascular endothelial cell-specific *RNF213* mutation, corresponding to the human p.Arg4810Lys variant, developed pulmonary hypertension after exposure to hypoxia [38], suggesting that environmental factors may trigger vasculopathy among susceptible individuals. Third, what determines which vessels of the body are affected? Why do some heterozygous patients develop PAH, whereas others develop moyamoya disease? We have reported on a family with the *RNF213* p.Arg4810Lys variant in which the mother had PAH and her daughter developed moyamoya disease [8]. Epigenetic or environmental factors may affect the vascular phenotypes. Fourth, is there a second genetic locus that defines organ-specific involvement when patients are heterozygous for *RNF213*? Fifth, are the *RNF213* pathogenetic variants associated with the venous involvement as well as the arterial phenotypes? Finally, this study was based on a retrospective analysis. Further prospective studies that emphasize the concept of *RNF213*-associated vascular disease are therefore needed.

In summary, several vasculopathies in different organs linked with *RNF213* variants can be integrated and organized within the concept of “*RNF213*-associated vascular disease”. Therefore, the genetic assessment of pathogenetic *RNF213* variants is essential when patients are diagnosed with rare vascular diseases. It is expected that the development of a diagnostic strategy and gene-associated treatment will effectively progress through the comprehensive capture of patients with multiple diseases on the basis of a common genetic background. It will be highly desirable to clarify all of the diseases in the *RNF213*-associated vascular disease category by means of global epidemiological investigation because of the possibility that such diseases may appear asymptomatically in some patients. Since the cause-and-effect relationships between *RNF213* variants and several vasculopathies have not been fully clarified, further studies are required to elucidate the underlying mechanisms and organ-specific factors that regulate the progression of *RNF213*-associated vascular disease.

## Figures and Tables

**Figure 1:**
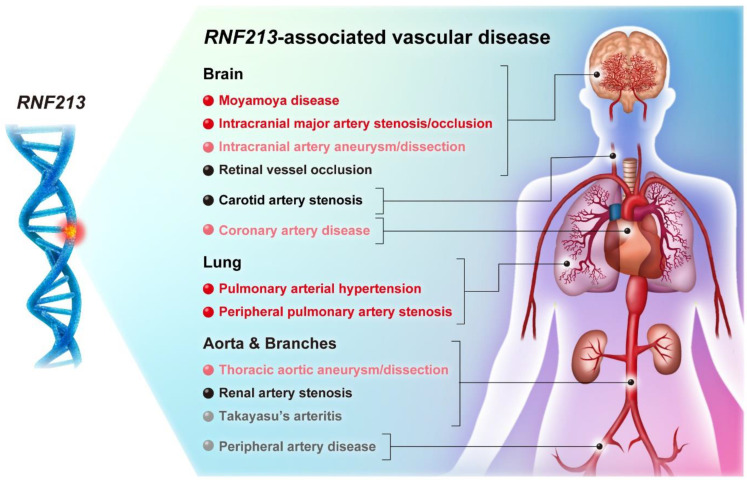
Conceptual diagram of “*RNF213*-associated vascular disease”. Pathogenetic variants of *RNF213* have been reported in several vasculopathies. Diseases with red letters (moyamoya disease, intracranial major artery stenosis/occlusion, pulmonary arterial hypertension, and peripheral pulmonary artery stenosis) indicate vasculopathies associated with the *RNF213* p.Arg4810Lys variant, which is a “hotspot” variant in the East Asian population. Pink letters (intracranial artery aneurysm/dissection, coronary artery disease, and thoracic aortic aneurysm/dissection) indicate diseases associated with *RNF213* variants. Black letters (retinal vessel occlusion, carotid artery stenosis, and renal artery stenosis) indicate diseases that are co-occurrent with moyamoya disease. Gray letters (Takayasu’s arteritis and peripheral artery disease) indicate diseases that may be associated with *RNF213* variants.
